# The Efficacy and Safety of Radiofrequency Ablation for Bilateral Papillary Thyroid Microcarcinoma

**DOI:** 10.3389/fendo.2021.663636

**Published:** 2021-06-11

**Authors:** Lin Yan, Mingbo Zhang, Qing Song, Jing Xiao, Ying Zhang, Yukun Luo

**Affiliations:** Department of Ultrasound, First Medical Center, Chinese PLA General Hospital, Beijing, China

**Keywords:** radiofrequency ablation, papillary thyroid carcinoma, thyroid, ultrasound, clinical outcomes

## Abstract

**Objective:**

To investigate the long-term clinical results of radiofrequency ablation (RFA) for bilateral papillary thyroid microcarcinoma (PTMC).

**Materials and Methods:**

From October 2014 to February 2018, 47 patients (37 females, 10 males, mean age 43.39 ± 9.26 years) with 100 bilateral PTMC (mean volume 75.22 ± 73.87 mm^3^) treated by RFA were included in this retrospective study. Bilateral PTMC was defined as at least one tumor located in the contralateral lobe. Patients were followed up at 1, 3, 6, 12 months and every 6–12 months thereafter. Volume, volume reduction ratio (VRR) and local tumor recurrence were evaluated during the follow-up period.

**Results:**

After a mean follow-up period of 47.77 ± 11.54 months, the mean volume of bilateral PTMC decreased from 75.22 ± 73.87 mm^3^ to 0.09 ± 0.44 mm^3^. The mean VRR was 99.94 ± 0.28% and the complete disappearance rate was 92.00%. During the follow-up, one patient (2.13%) developed lymph node metastasis and two patients (4.26%) had recurrent PTMC. All the recurrent lesions underwent additional RFA and two of them disappeared completely. No life-threatening or delayed complications occurred.

**Conclusions:**

With sufficient preoperative evaluation, RFA might be a promising alternative for bilateral PTMC patients who were unsuitable for surgery or refused surgery.

## Introduction

The incidence of thyroid cancer has increased worldwide, ranking in ninth among all cancers (1–4). Approximately 50% of the increase was attributed to the detection of papillary thyroid microcarcinoma (PTMC), which is a form of papillary thyroid cancer with a maximum diameter of 1 cm, with or without high-risk features (5). Bilateral lesions are very common in PTMC with an incidence of approximately 10–30% (6). It was previously correlated with a higher risk of locoregional recurrence. However, some studies have shown that tumor bilaterality was not associated with an increased risk of recurrence/persistence (7). According to the 8th AJCC/TNM risk of mortality system and American thyroid guideline (ATA) risk stratification, intrathyroidal bilateral PTMC was classified stage I with a low-risk of recurrence (8, 9). Surgery is the first-line treatment for bilateral PTMC. However, its drawbacks include invasiveness, cosmetic problems and life-long thyroid hormone replacement, which affect the quality of life (10). Moreover, the incidence of transient vocal fold paralysis and transient hypocalcemia after surgery were 5.5 and 4.3%, respectively (11). Radiofrequency ablation (RFA) as a minimally invasive technique, might be a potentially alternative for bilateral PTMC who were unsuitable for surgery or are contraindicated for surgery.

RFA is a commonly used thermal technique, has been reported as an effective and safe treatment for low-risk PTMC patients who refuse surgery or active surveillance (12–19). After ablation, the pooled proportion of volume reduction rate (VRR) was 98.1% (20) and the pooled proportion of complete disappearance rate was 57.6–76.2% (20, 21). However, the results were based mainly on the clinical outcomes of RFA for unifocal PTMC. To the best of our knowledge, no study has reported the long-term clinical outcomes of ablation for bilateral PTMC.

Therefore, the purpose of this study was to evaluate the efficacy and safety of RFA for bilateral PTMC.

## Materials and Methods

The Institutional Review Board of our institution approved this retrospective study. All the patients were provided written information consent before RFA. The RFA informed consent emphasized that surgery was the routine treatment recommended by guidelines.

### Patients

Bilateral PTMC was defined as at least one tumor located in the contralateral lobe (11). The inclusion criteria were: (1) PTMC lesions were confirmed by core-needle biopsy(CNB) or fine-needle aspiration(FNA); (2) no evidence of extrathyroidal extension(ETE) or lymph node(LN)/distant metastasis on US and chest CT; (3) patients who were unsuitable for surgery or refused surgery; (4) no neck irradiation history; (5) follow-up period was ≥24 months. The exclusion criteria of patients were: (1) no convincing evidence of aggressive disease by biopsy (9); (2) the maximum diameter of the tumor was ≥10 mm; (3) ETE was found; (4) LN metastasis or distant metastasis was detected; (5) patients with conscious disturbance or coagulation disorder or serious primary disease; (6) follow-up period was <24 months.

From October 2014 to February 2018, 468 patients with bilateral PTMC underwent treatment in this institution. Among them, 76 patients who were unsuitable for surgery or rejected surgery underwent RFA. Among them, patients with follow-up period less than 24 months (N = 29) were excluded. At last, a total of 47 patients with 100 low-risk PTMC were evaluated.

### Pre-Ablation Evaluation

Before RFA, patients all underwent thorough examinations, including complete blood count, thyroid function tests, coagulation tests and imaging evaluation, including ultrasound and chest CT (12–14). The volume of PTMC was calculated with the equations:

V=πabc / 6

V is the volume, while a is the largest diameter, b and c are the other two perpendicular diameters.

US were performed using a Siemens Acuson Sequoia 512 Ultrasound System (Siemens) or a Philips iU22 Ultrasound System (Philips Healthcare) or a Mindray M9 Ultrasound System (Mindray). CNB and RFA were all performed using a Siemens Acuson Sequoia 512 Ultrasound System. Contrast-enhanced ultrasound (CEUS) was used to assess the tumor before and immediately after RFA procedure. The ultrasound contrast agent was sulfur hexafluoride (SonoVueR). After injection of 2.4 ml SonoVue followed by a 5 ml of normal saline flush, CEUS was applied to observe the real-time microbubble perfusions within the tumor and the surrounding thyroid tissues.

### Ablation Procedures

All RFA procedures were performed by an experienced US physician. A bipolar RFA generator (CelonLabPOWER) and an 18-gauge bipolar RF applicator with 0.9 cm active tip (CelonProSurge micro 100-T09) were used.

Patients lay on an operating table in the supine position with the neck hyperextended. The targeted tumor was evaluated by multi-angle scanning to determine a practical and proper approach. Doppler ultrasound was used to access the detailed vascular anatomy along the approach route to prevent bleeding. Local anesthetic (1% lidocaine) was injected at the subcutaneous puncture site and the thyroid anterior capsule. During the RFA procedure, the smaller lesion was ablated first.

RFA procedure was performed using trans-isthmic approach and moving-shot technique. To prevent thermal injury, hydrodissection technique was performed by injection of normal saline to separate the target tumor from critical structures (trachea, carotid artery, jugular vein, esophagus and recurrent laryngeal nerve). Normal saline was injected using another needle (23 gauge) to form at least 1 cm distance between the tumor and the critical structure (13, 14).

The initial RFA power was 3 W and was increased to 5 to 9 W if a transient hyperechoic zone did not form at the electrode tip in 10 s. To prevent marginal residue and recurrence, we enlarged the ablation area which exceeded the tumor edge (at least 3–5 mm) (21, 22). CEUS was performed immediately after the RFA procedure to assess the ablation area. If any enhancement existed, a complementary RFA should be applied.

### Post-Ablation Assessment

Patients were followed up at 1, 3, 6 months and every 6–12 months thereafter by US, CEUS and chest CT. At 3 or 6 months after RFA, the ablated area was evaluated by CNB, which was performed to the central zone, the peripheral zone and surrounding thyroid parenchyma (22–24). The development of metastatic LNs and the suspicious new lesions were submitted to biopsy. The volume reduction rate (VRR) was calculated by the equations:

$$\text{VRR}=\frac{\left(\text{initial volume}-\text{final volume}\right)}{\text{initial volume}}\times 100\%$$

RFA was considered to successful when one of the criteria were met (25): (1) the ablated tumor was completely disappearance; (2) the ablated tumor remained as scar-like on US but absence of enhancement on both arterial and venous phase on CEUS; (3) If the ablation area still existed, no malignant cells was confirmed by CNB. Local tumor recurrence was defined to include two situations (26): (1) new lesion was confirmed to be PTMC after CNB; (2) cervical LN metastasis was confirmed after biopsy. Distant metastasis was diagnosed by CT, positron emission tomography or bone scan when there were suspicious symptoms. Delayed surgery is defined as that patients received surgery due to tumor progression or anxiety during the follow-up.

### Statistical Analysis

Statistical analysis was performed using the SPSS statistical software (Version 25.0). Continuous data were presented as mean ± SD (range). Non-parametric Wilcoxon signed-rank tests were used to compare the pre-ablation with post-ablation volume as each follow-up period. A *P <*0.05 was considered as statistically significant.

## Results

Clinical characteristics of patients before RFA are shown in **Table 1**. Among the 47 patients, 41 had two tumors and six had three tumors. The mean diameter was 4.82 ± 1.57 mm and the mean volume was 75.22 ± 73.87 mm^3^. The volume of dominant tumor was 108.77 ± 87.08 mm^3^.

**Table 1:** Clinical characteristics of patients.

Characteristics	Data
No. of patients	47
No. of tumors	100
Patients with two tumors, n (%)	41 (87.23)
Patients with three tumors, n (%)	6 (12.77)
Age (years)	43.39 ± 9.26 (23–63)
Female (%)	37 (78.72)
Mean diameter (mm)	4.81 ± 1.57 (0.20–0.93)
Mean Volume (mm^3^)	75.22 ± 73.87 (4.19–424.10)
Volume of dominant tumor (mm^3^)	108.77 ± 87.08 (8.12–424.10)

Data are expressed as mean ± SD (range).

For each tumor, the mean power was 3.92 ± 0.98 W. The mean RFA time was 224.09 ± 156.68 s and the mean energy was 853.64 ± 614.39 J.

### Efficacy

**Figure 1:**
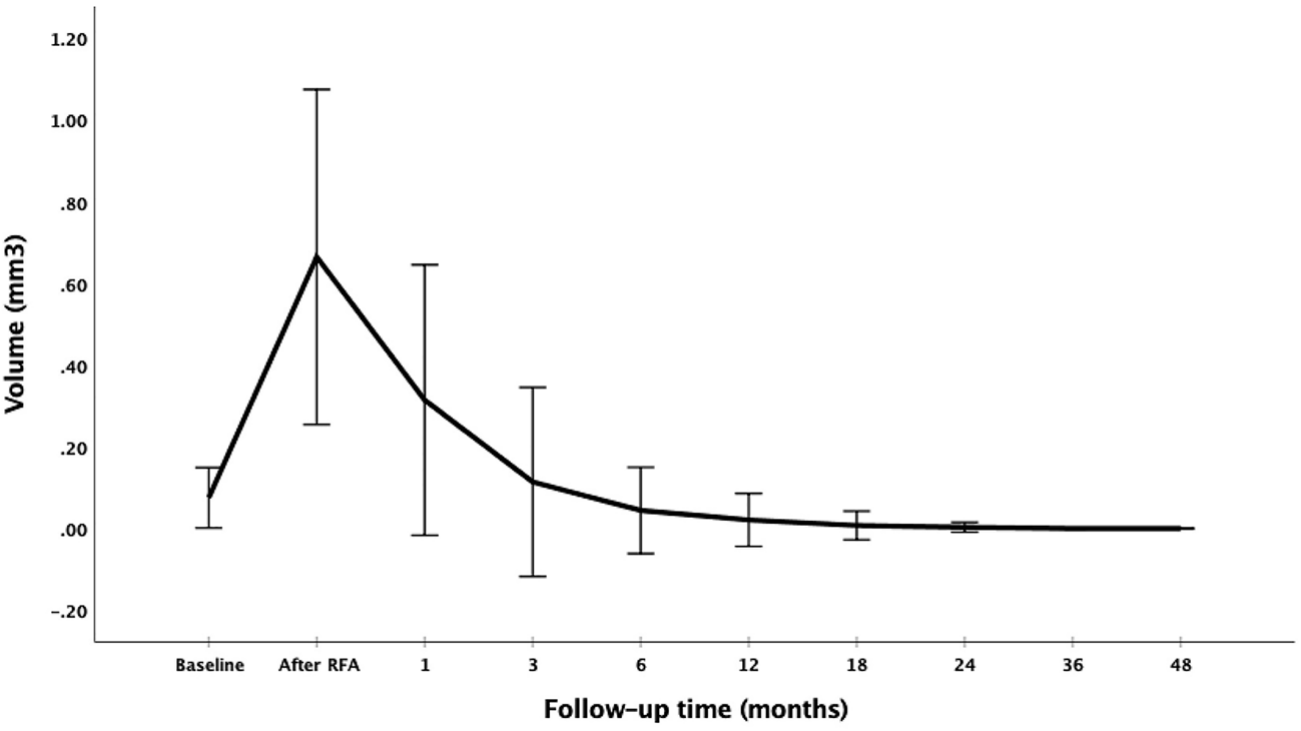
Changes of volume at each follow-up period after RFA.

**Figure 2:**
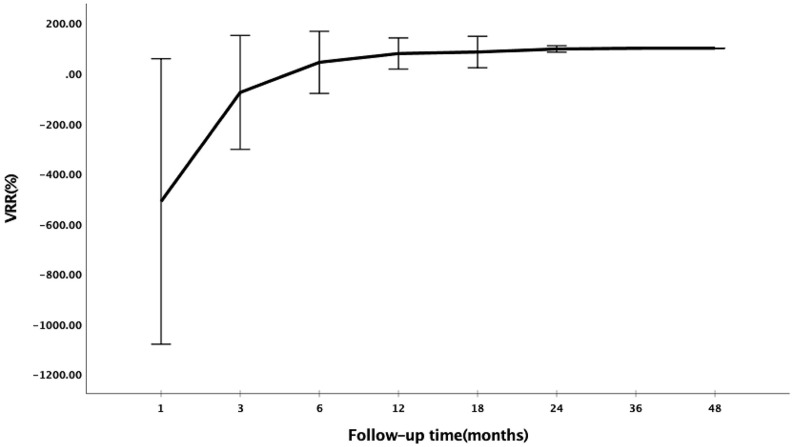
Changes of VRR at each follow-up period after RFA.

**Figure 3:**
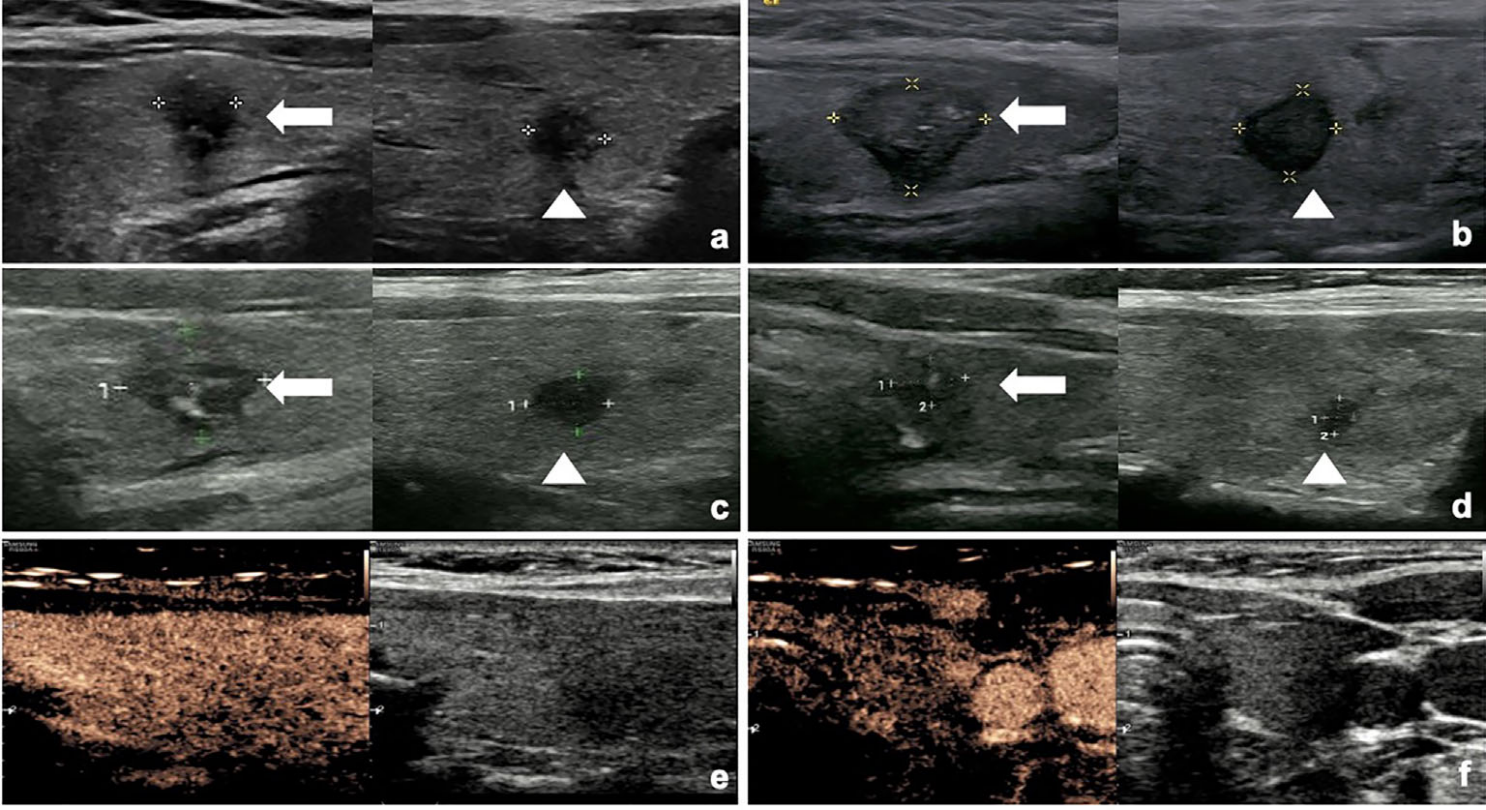
The US and CEUS images of a 32-year-old female with bilateral PTMC during the follow-up. **(A)** Before RFA, two PTMC lesions were confirmed by CNB. One tumor was located in the left lobe with an initial volume of 78.54 mm^3^ (arrow) and the other one was in right lobe with an initial volume of 65.45mm^3^ (arrowhead). **(B)** At 1 month after RFA, the volume of left tumor (arrow) was 898.47 mm^3^ and the volume of right tumor (arrowhead) was 234.57 mm^3^. **(C)** At 3 months after RFA, the volume of left tumor (arrow) was 226.19 mm^3^ and the volume of right tumor (arrowhead) was 78.54 mm^3^. **(D)** At 6 months after RFA, the volume of left tumor (arrow) was 50.26 mm^3^ and the volume of right tumor (arrowhead) was 12.57 mm^3^. **(E, F)** At 12 months after RFA, these two ablated tumors both disappeared.

The changes of volume and VRR after RFA at each follow-up visit are presented in **Table 2**. The mean follow-up period was 47.77 ± 11.54 months (range 24–76 months). The mean volume of the ablation areas was significantly larger than the initial volume at first 3 months (*P <*0.001) because of the enlarge ablation, which was gradually decreased from 6 months after RFA (**Figure 1**). The mean VRR was 99.94 ± 0.28% (**Figure 2**) and the overall complete disappearance rate was 92.00% (92/100). The numbers of completely disappearance were 1 (1.00%), 12 (12.00%), 21 (21.00%), 26 (26.00%), 18 (18.00%), 10 (10.00%) and 4 (4.00%) at 1, 3, 6, 12, 18, 24, and 36 months after RFA, respectively. A representative case underwent RFA is presented in **Figure 3**.

**Table 2:** Changes of the volume and VRR after RFA at each follow-up.

Time	Volume (mm^3^)		*p* value (*Vs* initial volume)	VRR (%)	
	Mean ± SD	range		Mean ± SD	range
initial	75.22 ± 73.87	4.19–424.10	–	NA	
After RFA	664.13 ± 410.00	109.95–1,858.20	<0.001	NA	
1 month	314.35 ± 331.09	10.47–2,205.33	<0.001	−511.78 ± 569.06	−2,600–100
3 months	114.02 ± 231.45	0–1,910.03	<0.001	−76.40 ± 227.17	−985.71–100
6 months	44.31 ± 105.58	0–680.66	<0.001	43.01 ± 123.00	−585.71–100
12 months	21.11 ± 64.77	0–376.98	<0.001	77.85 ± 62.28	−255.56–100
18 months	9.76 ± 37.86	0–241.90	<0.001	84.97 ± 62.85	−350.00–100
24 months	3.29 ± 11.97	0–75.40	<0.001	96.97 ± 12.84	3.57–100
36 months	0.25 ± 1.42	0–9.42	<0.001	98.78 ± 1.28	91.43–100
48 months	0.09 ± 0.44	0–2.09	<0.001	99.94 ± 0.28	98.64–100

NA, not applicable.

### Local Tumor Recurrence

Because 13 tumors disappeared in the first 3 months and another six tumors disappeared at 6 months, a total of 81 tumors underwent post-ablation CNB and the results were all negative.

The incidence of LN metastasis and of recurrence PTMC was 2.13% (1/47) and 4.26% (2/47), respectively. No distant metastasis was detected. No patient underwent delayed surgery during the follow-up. The clinical characteristics and outcomes of PTMC patients with local tumor recurrence are showed in **Table 3**. One patient developed LN metastasis in the central compartments at 12 months after RFA with a volume of 100.53 mm^3^. Two patients had recurrent PTMC and the volume was 18.85 mm^3^ and 153.93 mm^3^, respectively. All of these three patients underwent additional RFA. Two recurrent PTMCs were completely disappeared during the follow-up. The metastatic LN shrunk to 37.70 mm^3^ after 1-year follow-up. This lesion underwent CNB and demonstrated no malignancy.

**Table 3:** The clinical characteristics and outcomes of patients with local tumor recurrence.

	No. of patients	Sex/age	No. of PTMC lesions	Dominant tumor Location/Volume (mm^3^)	Developed Time(months)	Location	Volume(mm^3^)	Treatment	Outcomes
LN metastasis (N = 1)	1	F/34	2	Isthmus/150.79	12	Right, Level VI	100.53	RFA	37.70^a^
Recurrent PTMC (N = 2)	1	F/43	2	Left/43.33	6	Right lobe	18.85	RFA	Disappeared at 12-month after additional RFA
	2	F/32	2	Right/53.33	12	Right lobe	153.93	RFA	Disappeared at 12-month after additional RFA

a Data are represented as the volume at last follow-up (mm^3^).

### Safety

All the 47 patients were tolerable to RFA. Four patients underwent local pain or discomfort and they all resolved spontaneously within 3 days. No patients had major or delayed complications.

## Discussion

To date, no study has been evaluated the clinical application of RFA for bilateral PTMC. In this study, after a mean follow-up period of 47.77 ± 11.54 months, the mean volume of bilateral PTMC decreased from 75.36 ± 73.86 mm^3^ to 0.09 ± 0.43 mm^3^ with a mean VRR of 99.94 ± 0.28%. A total of 92 tumors disappeared completely. The incidences of LN metastasis and of recurrent PTMC were 2.13 and 4.26%, respectively. All recurrent lesions were successfully treated with an additional RFA. No major complications or sequelae were observed.

Bilaterality has been observed in approximately 10–30% of patients with PTMC (6). It refers to the presence of multiple synchronous primary tumors, arising from independent clones, instead of intraglandular metastasis from a single primary tumor *via* intraglandular lymphatics (27, 28). However, the prognostic value of bilateral PTMC remains controversial. Studies recommended more aggressive treatments such as total thyroidectomy with central neck dissection and subsequent radioactive iodine ablation therapy for bilateral PTMC, which has an increased loco-regional recurrence rate (29, 30). In contrast, Choi et al. (7) found that bilateral PTMC was not an important prognostic factor in PTMC patients, but in the non-PTMC patients. Zhou et al. (6) also reported that bilateral PTMC was significantly associated with central LN metastasis on the univariate analysis. However, this was not an independent risk factor after multivariate logistic regression analysis. The conflicting results were likely due to the early detection and treatment of bilateral lesions, which did not fully exhibit its biological behavior.

Although thermal ablation is not the first-line treatments for PTMC, it has been applied to unifocal low-risk PTMC patients concerned about active surveillance or complications after surgery (17, 25, 31–38). A few patients with multifocal or bilateral PTMC were also treated with ablation, but the clinical outcomes were not reported separately (14–16, 39). The viability of RFA as an alternative for bilateral PTMC patients who refuse surgery or are unsuitable for surgery is unknown. This study included 100 bilateral PTMC lesions in 47 patients who were unsuitable for surgery or refused surgery. This was the first study to evaluate the clinical results of RFA for bilateral PTMC. After a mean follow-up period of 47.77 ± 11.54 months, the VRR was 99.94 ± 0.28% and 92% of tumors completely disappeared. This was consistent with previous studies on RFA for unifocal PTMC, which reported a VRR of 90–100%, with a complete disappearance rate of 15.22–100% (17, 25, 31–38). This demonstrated that the efficacy of RFA for bilateral PTMC was comparable to that of unifocal PTMC.

In terms of safety, a meta-analysis reported that the pooled proportion of overall complications after thermal ablation was 3.1% and major complications occurred in 0.7% (20). The pooled proportion of complications after RFA was 1.7% (21). In this study, only four patients experienced local pain or discomfort. None of the patients experienced major complications. Several strategies were related to the low incidence of complications. First, RFA was performed by an experienced physician. A detailed preoperative evaluation and adequate knowledge of neck anatomy on US could minimize the incidence of complication (40). Second, during the RFA procedure, smaller lesion was usually treated first and the needle tip was monitored continuously and cautiously *via* US. Third, safe precautions against thermal injuries, such as the moving-shot technique, trans-isthmic approach and hydrodissection technique, were also performed during the RFA procedure (40).

The local tumor recurrence rate after ablation for unifocal PTMC is low. A previous study showed that the incidence of LN metastasis and recurrent PTMC following unifocal PTMC ablation were 0.84–2.98 and 1.19–2.78%, respectively (25, 34–38). A similar incidence of LN metastasis (2.13%) was observed in this study. However, the incidence of recurrent PTMC for bilateral PTMC was 4.26%, which was higher than that for unifocal PTMC. This was likely due to the low sensitivity of US in detecting multiple tumors. Thus, occult tumor foci may have been missed by preoperative evaluation. Although all recurrent lesions were successfully treated by additional RFA, sufficient preoperative evaluation was crucial in identifying the number and location of tumors and formulating an appropriate treatment strategy. Surgery remains the first-line treatment for bilateral PTMC. However, patients who refuse or are ineligible for surgery can opt for RFA for palliative purposes, with fully informed consent and thorough follow-up management.

This study had some limitations. First, it was a single-center retrospective study. Second, the sample size of bilateral PTMC cases was relatively small. Third, the follow-up period was relatively short. Given the good prognosis of PTMC, further studies with a larger number of bilateral PTMC patients and a more extended follow-up period are needed. Fourth, this study did not compare RFA with surgery for the treatment of bilateral PTMC.

In conclusion, with sufficient preoperative evaluation, RFA might be a promising treatment for bilateral PTMC patients who were unsuitable for surgery or refused surgery.

## Data Availability Statement

The raw data supporting the conclusions of this article will be made available by the authors, without undue reservation.

## Ethics Statement

The studies involving human participants were reviewed and approved by The Institutional Review Board of Chinese PLA General Hospital. The patients/participants provided their written informed consent to participate in this study.

## Author Contributions

LY interpreted the patient data and drafted the manuscript. YKL performed RFA procedure, and conceived of the study and coordination. MBZ, QS, JX, and YZ collected and analyzed the patient data. All authors contributed to the article and approved the submitted version.

## Funding

This study is supported by Beijing Municipal Science & Technology Commission (No. Z181100001718017) and the Research of Healthcare Big Data of Chinese PLA General Hospital (2019MBD-040).

## Conflict of Interest

The authors declare that the research was conducted in the absence of any commercial or financial relationships that could be construed as a potential conflict of interest.
